# Proposing FDA consideration for the treatment and prophylaxis of opioid and psychostimulant abuse to incorporate the induction of DNA guided dopamine homeostasis: Anti-reward deficiency restoration solution (ARDS)

**DOI:** 10.15761/JSIN.1000253

**Published:** 2020-12-15

**Authors:** Kenneth Blum, John Giordano, David Baron, Thomas McLaughlin, Rajendra D Badgaiyan

**Affiliations:** 1 Western University Health Sciences, Graduate College, Pomona CA, USA; 2 The Kenneth Blum Behavioral & Neurogenetic Institute (Division of Ivitalize) Austin, TX, USA; 3 Department of Psychiatry, South Texas Veteran Health Care System, Audie L. Murphy Memorial VA Hospital, San Antonio, TX, Long School of Medicine, University of Texas Medical Center, San Antonio, TX, USA

**Keywords:** FDA, anti-reward deficiency restoration solution (ards), DNA polymorphisms, dopamine homeostasis, pro -dopamine regulation, kb220, nutrigenomics

## Abstract

**Background:**

In face of an American opioid/psychostimulant crisis with overdose fatalities, due, in part, to the COVOD 19 pandemic, we are proposing a paradigm shift in response. Currently, The FDA has approved pharmaceuticals for the treatment of opioids, alcohol, and nicotine but not for psychostimulants or even cannabis.

**Proposition:**

To respond to the deadly overdose issue globally, we are proposing that the FDA embrace, for the treatment and prophylaxis of opioid and psychostimulant abuse, induction of DNA-guided, dopamine homeostasis. We refer to this novel therapeutic target as the Anti-Reward Deficiency Restoration Solution (ARDS).

**Expert opinion:**

This futuristic proposal regarding the FDA will provide important information that may ultimately lead to significant improvement in the recovery of individuals with opioid/psychostimulant and polydrug abuse issues, especially, those with genetically-induced dopamine deficiency.

**Conclusion:**

With large populations supporting these initial results, and possibly even additional candidate genes and single nucleotide polymorphisms, the neuroscience and neurological community may eventually have the clinical ability to classify addiction severity, according to genotype and possession of risk alleles. A promising goal is the identification of high risk vulnerability, along with the provision of a safe, non-addicting ARDS natural nutrigenomic, involving a therapeutic model that potentially up-regulates instead of down-regulates dopaminergic receptors, preferably, the D2 subtype, is one laudable goal.

## Introduction

The now, well-characterized opioid epidemic is a primary public global health concern [1]. In the United States alone, at least 100 people are dying from opiate/opioid overdose every day. According to recent polls taken in America, this epidemic exceeds the concerns of even terroristic threats, and its prevention is tantamount to any successful vaccination program targeted against COVID 19 [2]. Moreover, there is also an increase abuse of other psychoactive substances, such as, alcohol and psychostimulants during the COVID 19 pandemic [3].

From this perspective, we encourage the neuroscience and neurological community to focus on both genetic polymorphisms of many reward genes and their epigenetic modifications leading to vulnerability and or resistance to use and misuse of opiates/opioid to treat pain [4]. We point out that a universal goal in combating the unwanted opioid crisis, especially, in America, is to trace the neurochemical mechanisms of acute opiate withdrawal and use alpha 2 antagonists, like clonidine, as well as specific target loci, followed by the understanding of opiate/opioid reward mechanisms [5].

We point out here and in many previous publications from our group as well as others, the alteration of brain functional connectivity based on neurobiological mechanisms seems to be an important therapeutic target [6,7]. It is well known that, for example, animal models of heroin dependence depict the important role of disrupted fronto-striatal circuitry supporting cognitive control processes and even heroin-seeking [8]. Along these lines, Qui *et al.* [9], investigated the corpus callosum (CC), known to connect homologous regions of the cortex, and, as such, is the major gateway for information transfer between the cerebral hemispheres and represents a structural connectivity index between hemispheres. Qui’s results reveal that there exists a substantial deficit of interhemispheric coordination in patients with heroin dependence. Moreover, interhemispheric connectivity was shown to be correlated with the duration of heroin abuse and higher impulsivity behavior. In agreement with this work, Li *et al.* [10] also showed structural and functional connectivity within the default mode network (DMN) are both disturbed in heroin dependent people. This disturbance progresses as duration of heroin use escalates and is associated with deficits in decision making in heroin addiction. It is noteworthy that Xu *et al.* [11] in healthy individuals, found a significant non-additive *COMT* × *DRD2* interaction in the right dorsal anterior cingulate cortex (dACC), exhibiting an inverted U-shape modulation by dopamine signaling. In agreement, Lachowicz *et al.* [12], recently reported a haplotype association consisting of five polymorphisms in the *DRD2/ANKK1* region: rs1076560, rs1800498, rs1079597, rs6276 in the *DRD2* gene, and rs1800497 in the *ANKK1* gene that favored relapse, compared to non -risk allelic controls. These data suggest a hypodopaminergic trait impacted by epigenetics. While there is a paucity of information related to a direct epigenetic impact on opioids (e. g. morphine and heroin) work from DiNieri *et al.* [13], revealed that maternal cannabis abuse alters developmental regulation of mesolimbic DRD2 in offspring through epigenetic mechanisms that regulate histone lysine methylation, and the ensuing reduction of DRD2 could contribute to addiction to opioid vulnerability later in life. Earlier work from Dalterio *et al.* [14], agrees with this later study, showing that perinatal exposure to delta 9-THC in mice altered enkephalin and norepinephrine sensitivity in vas deferens, with possible future life vulnerability to opioid type drugs. This has been further confirmed by additional work by Blum *et al.* [15], showing that rodent genotype dictates sensitivity to normorphine on the vas deferens.

Along similar lines, Maze *et al.* [16], also revealed that repression of histone methyltransferase (HMT) G9a by chronic cocaine administration occurs in both Drd1-expressing (striatonigral) and Drd2-expressing (striatopallidal) medium spiny neurons. This work points to the role of epigenetics in regulating expression in doapminergic genes and suggests a critical function for cell type-specific histone methylation patterns in the regulation of behavioral responses to environmental stimuli.

One area that requires intensive investigation involves psychostimulant abuse, since there is no approved FDA therapeutic for this syndrome. Dysregulated striatal-cortical network interactions have been identified in cocaine addiction. Specifically, research from NIDA and Stein’s group [17] reported enhanced rsFC strength, predominantly in striatal-frontal circuits; reduced rsFC observed between the striatum and cingulate, striatal, temporal, hippocampal/amygdala, and insular regions in the cocaine group, compared with the non -drug abusing controls [7]. In addition, Hu *et al.* [17] found an augmented striatal-dorsal lateral prefrontal cortex connectivity which was positively correlated with the amount of recent cocaine use and as elevated trait of impulsivity. Specifically, an index reflecting the balance between striatal-dorsal anterior cingulate cortex and striatal-anterior prefrontal/orbitofrontal cortex circuits was associated with loss of control over cocaine use. This work and other published reports [18] suggest that cocaine dependence is associated with disturbed rsFC in several specific striatal-cortical circuits. This understanding coupled with genetic reward gene antecedents like the association of the DRD2 A1 allele and cocaine dependence [19], strongly suggest that any FDA-approved drug for Psychostimulant Use Disorder (PUD) must consider induction of “dopamine homeostasis”.

One important clinical application for this approval process from the FDA is linked to the abuse of methylphenidate in the ADHD community. Badgaiyan *et al.*, utilized a unique, dynamic molecular imaging technique [20] to access real -time dopamine release. PET data were analyzed to measure dynamic changes in ligand binding potential (BP) and other receptor kinetic parameters. Their analysis revealed that, at rest the ligand BP was significantly higher in the right caudate of ADHD volunteers, suggesting reduced tonic release. However, during task performance they observed a significantly lower ligand BP, indicating increased phasic release. Badgaiyan *et al.* pointed out that ADHD tonic release of dopamine reflects a hypodopaminergia but that, in contrast, the phasic release is enhanced in the right caudate [21]. These data characterize a lowered dopamine trait in ADHD, potentially genetic in origin but which may be overcome through physiologic induction of, possibly, epigenetic induced hyperdopaminergia as a state response. Moreover, Konova *et al.* [22], reported that short-term, methylphenidate administration decreased an abnormally strong connectivity of the ventral striatum with the dorsal striatum (putamen/globus pallidus). Furthermore, they observed an attenuated connectivity between these regions during placebo administration, uniquely correlated with less severe addiction. Of interest and in contrast, methylphenidate strengthened several cortico-limbic and cortico-cortical connections. This later finding suggests that the effects of methylphenidate within striatal and cortical pathways constitute a potentially viable mechanism by which methylphenidate might facilitate control of behavior in cocaine dependence.

The take home message here is that, based on this data, FDA approval should consider the role of dopamine in psychostimulant abuse and the required need for stabilization or balance of at least dopaminergic brain activity [23].

Moreover, we provide a novel approach, whereby, instead of just blocking acute withdrawal symptoms as indicated in the DSM –V, we propose that at the onset of detoxification, clinicians should genetically diagnose each patient to not only determine risk stratification but determine polymorphic targets for either pharmaceutical or nutraceutical intervention like glutaminergic-dopaminergic optimization complex (GDOC) [24,25].

In this regard, we are proposing a novel Genetic Addiction Risk Severity (GARS) test that could be coupled with the nutraceutical, KB220 variant, which is precision-matched to the addict’s brain polymorphisms across the Brain Reward Cascade (BRC).

It is now well accepted that dopaminergic function at the Ventral Tegmental Area (VTA) and subsequent release of dopamine at the N. Accumbens (NAc), varies widely but is a significant protracted alteration even in abstinent, heroin addicts [26,27] and psychostimulant abusers. As such, we are proposing a new “anti-reward deficiency restoration solution” (ARDS), whereby, instead of just blocking withdrawal symptoms, utilizing powerful opioids to treat Opioid Use Disorder) (OUD) (harm reduction), for example, clonidine, in combination with methadone/buprenorphine/ naloxone. We are instead proposing the embracing of gentile. dopaminergic, agonistic therapy (possibly, though optogenetic stimulation) as a preferred modality, initiated early in recovery [28] (at detoxification).

In terms of OUD, there is emerging evidence related to augmented rsFC (resting state functional connectivity) in abstinent OUD patients. As a result, our laboratory [29], evaluated the effect of KB220Z on reward circuitry of 10 heroin addicts undergoing protracted abstinence (average =16.9 months). In a randomized, placebo-controlled, crossover study of KB220Z, five subjects completed a triple-blinded experiment. In addition, nine subjects were genotyped utilizing the GARS test. We found that KB220Z induced an increase in BOLD activation in caudate-accumbens-dopaminergic pathways, compared to placebo, following 1-hour acute administration. KB220Z also reduced a hyperdopaminergic state resting-state activity in the cerebellum of abstinent heroin addicts. In the second phase of this pilot study, of all 10 abstinent heroin-dependent subjects, our group found that three brain regions of interest were significantly activated from resting state by KB220Z compared to placebo. Enhanced functional connectivity occurred in a putative network that included the dorsal anterior cingulate, medial frontal gyrus, nucleus accumbens, posterior cingulate, occipital cortical areas, and cerebellum. These results and other quantitative electroencephalogy (qEEG) study results suggest a putative, anti-craving/anti-relapse role of KB220Z, in addiction by direct or indirect dopaminergic interaction.

Willuhn *et al.* [30] reported that cocaine use and even non-substance-related addictive behavior increases as dopaminergic function is reduced. Chronic cocaine exposure has been associated with reductions in D2/D3 receptors and associated with attenuated activation of cues in occipital cortex and cerebellum in a recent PET study by Volkow *et al.* [31]. Indeed, treatment strategies, like dopamine agonist therapy, that might conserve dopamine function may be an effective therapeutic to relapse prevention in psychoactive drug and behavioral dependencies. In terms of Psychostimulant abuse, Blum *et al.* [32], have reported that positive outcomes, demonstrated by quantitative electroencephalographic (qEEG) imaging in a randomized, triple-blind, placebo-controlled, crossover study, involving oral KB220Z^™^ showed an increase of alpha waves and low beta wave activity in the parietal brain region. With *t* statistics, differences observed between placebo and KB220Z consistently occurred in the frontal regions after week 1 and then, again, after week 2 of analyses. This was the first report to demonstrate involvement of the prefrontal cortex in the qEEG response to a natural putative D2 agonist (KB220Z), especially, evident in dopamine D2 A1 allele subjects. It is noteworthy that these results are indicative of a phase change from low amplitude or low power in the brain to a more regulated state by increasing an average of 6.169 mV (2) across the prefrontal cortical region, suggesting dopamine homeostasis.

Our proposed ARDS model is based on known mechanisms, involving serotonergic, endorphinergic, cannabinergic, glutaminergic, cholinergic and dopaminergic pharmacology, leading to the long-term development of “dopamine homeostasis” in order to treat and, even prevent, future relapse of opiate/opioid/psychostimulant use or misuse [33]. We term this novel therapy “Precision Addiction Management (PAM) [34]. It should be noted as an important caveat in the field of Psychiatric Genetics, there is a paucity of studies linked to ethnic differences in terms of gene polymorphisms and potential vulnerability to reward deficiency behaviors [35].

## Conclusion

This proposal concerning the FDA is written in the spirit of just providing important information that may ultimately lead to significant improvement in the recovery of individuals with opioid/psychostimulant and polydrug abuse issues, especially, those with genetically induced dopamine deficiency [36].

We are proposing that, with the use of necessary large populations to support these initial results, and, possibly, the use of even additional candidate genes and single nucleotide polymorphisms, the neuroscience and neurological community may eventually have the clinical ability to classify severity according to genotype and possession of risk alleles. This classification may then be combined with a safe, non-addicting, ARDS natural-targeted system and integrative nutrigenomic therapeutic model that upregulates, instead of downregulating dopaminergic receptors, preferably, the D2 subtype [37]. However, the goal is to achieve “dopamine homeostasis”.
